# Ultrasound in addition to clinical assessment of acute musculoskeletal complaints in bleeding disorders: impact on patient management

**DOI:** 10.1016/j.rpth.2024.102372

**Published:** 2024-03-15

**Authors:** Flora Hendrica Pieternella van Leeuwen, Wouter Foppen, Pim A. de Jong, Wobke E.M. van Dijk, Johan Blokzijl, Kathelijn Fischer, Merel A. Timmer

**Affiliations:** 1 Department of Radiology and Nuclear Medicine, Division of Imaging & Oncology, University Medical Center Utrecht, Utrecht University, Utrecht, the Netherlands; 2 Center for Benign Haematology, Thrombosis and Haemostasis, Van Creveldkliniek, University Medical Center Utrecht, Utrecht University, Utrecht, the Netherlands

**Keywords:** hemarthrosis, hemophilia, physical examination, point-of-care testing, ultrasonography

## Abstract

**Background:**

Ultrasound is increasingly used for musculoskeletal assessment in hemophilia care.

**Objectives:**

To evaluate the impact of point-of-care ultrasound added to clinical assessment for diagnosis and treatment of acute musculoskeletal episodes in a heterogeneous cohort of children and adults with hemophilia and von Willebrand disease (VWD).

**Methods:**

This prospective cross-sectional study consecutively included children and adults with hemophilia or VWD who visited the outpatient clinic with acute musculoskeletal complaints between March 2020 and May 2023. For all episodes, initial diagnosis and treatment determined by clinical assessment were recorded on a case report form. Subsequently, a physiotherapist (M.A.T. and J.B.) with knowledge of the clinical diagnosis performed point-of-care ultrasound. After ultrasound, updated diagnosis and treatment were recorded. Diagnosis and treatment before and after ultrasound were compared, and proportions of change with 95% CIs were determined.

**Results:**

We evaluated 77 episodes in 67 patients (median age, 24 years; IQR, 13-42 years). Before ultrasound, 37 joint bleeds, 13 muscle bleeds, and 27 other diagnoses were diagnosed. After ultrasound, 33 joint bleeds, 11 muscle bleeds, and 33 other diagnoses were confirmed. The diagnosis changed in 28 of 77 episodes (36%; 95% CI, 26%-48%). Nine joint bleeds and 2 muscle bleeds were missed by clinical assessment. Ultrasound findings changed treatment strategy in 30 of 77 episodes (39%; 95% CI, 28%-51%).

**Conclusion:**

Ultrasound in addition to clinical assessment of acute musculoskeletal complaints in people with hemophilia and VWD has an impact on diagnosis (36%) and treatment (39%), which supports the use of ultrasound in acute musculoskeletal complaints in hemophilia and VWD.

## Essentials

• Ultrasound may impact management of acute musculoskeletal episodes in bleeding disorders.
• Children and adults with musculoskeletal episodes were assessed clinically and with ultrasound.
• Ultrasound changed the diagnosis in 36% and treatment in 39% of the 77 musculoskeletal episodes.
• The number needed to scan with ultrasound to achieve diagnostic/therapeutic impact was 3.

## 1 Introduction

Hemarthrosis and intramuscular bleeding are characteristic for hemophilia [1] and occur to a lesser extent in von Willebrand disease (VWD) [2]. Approximately 60% to 80% of bleeding in people with hemophilia occurs in the joints, followed by 10% to 30% in muscles [3,4]. Both joint and muscle bleeds cause acute pain, swelling, and reduced function [5]. Furthermore, intra-articular blood damages the joint in a multifactorial way. Recurrent joint bleeding can ultimately lead to irreversible and invalidating joint damage known as hemophilic arthropathy [1,[6], [7], [8]]. Muscle bleeding that is not well managed can lead to compartment syndrome, muscle contracture, and necrosis [3]. In rare cases, muscle bleeding can be complicated by pseudotumor formation [9,10].

In the acute phase, joint and muscle bleeds are treated with clotting factor concentrate to stop the bleeding, prevent rebleeding, and prevent the progression to either hemophilic arthropathy or permanent muscle contractures and pseudotumor formation. When the bleeding has stopped, the comprehensive treatment focuses on functional rehabilitation [3].

Joint and muscle bleeds are usually diagnosed and treated based on clinical assessment. However, clinical symptoms of musculoskeletal bleeding, such as pain, swelling, and limited range of motion, are not specific to bleeding episodes [[11], [12], [13], [14]]. Especially the differentiation between a hemarthrosis and a painful arthropathy flare-up can be difficult because of overlapping symptoms [11]. As treatment for bleeding episodes and arthropathy flare-ups/other musculoskeletal diagnoses is different, it is important to accurately distinguish between these diagnoses.

Ultrasound is increasingly used to evaluate joint health in hemophilia care [[15], [16], [17]]. It is a fast, noninvasive, relatively inexpensive, and accurate modality to assess blood-related changes in the musculoskeletal system with good reproducibility [[18], [19], [20], [21]]. Ultrasound can also be used in acute settings as it is sensitive for detecting joint and muscle bleeding [3,14,22,23]. Most diagnostic studies assess differences between diseased and nondiseased, and some test diagnostic accuracy. However, for ultrasound to be useful when added to clinical assessment, it needs to impact diagnosis and treatment. Evidence on the diagnostic and therapeutic impact of ultrasound in acute musculoskeletal episodes in bleeding disorders is limited. Three previous studies in adult patients, the majority of whom had preexisting joint damage, have reported discrepancies between clinical diagnosis and ultrasound findings in 16% to 65% of painful musculoskeletal episodes, indicating an added diagnostic value of ultrasound [14,24,25]. It remains unknown whether clinical misdiagnosis occurs as frequently in a more heterogeneous and younger patient population.

In this cross-sectional study, we evaluated the diagnostic and therapeutic impact of point-of-care ultrasound in addition to clinical assessment in a heterogeneous cohort of children and adults with hemophilia or VWD, with and without preexisting joint damage, who presented with an acute musculoskeletal episode. Our secondary aim was to explore if the clinical symptoms suggested in previous studies [26,27] can identify joint bleeding.

## 2 Methods

### 2.1 Study design and population

**Figure 1:**
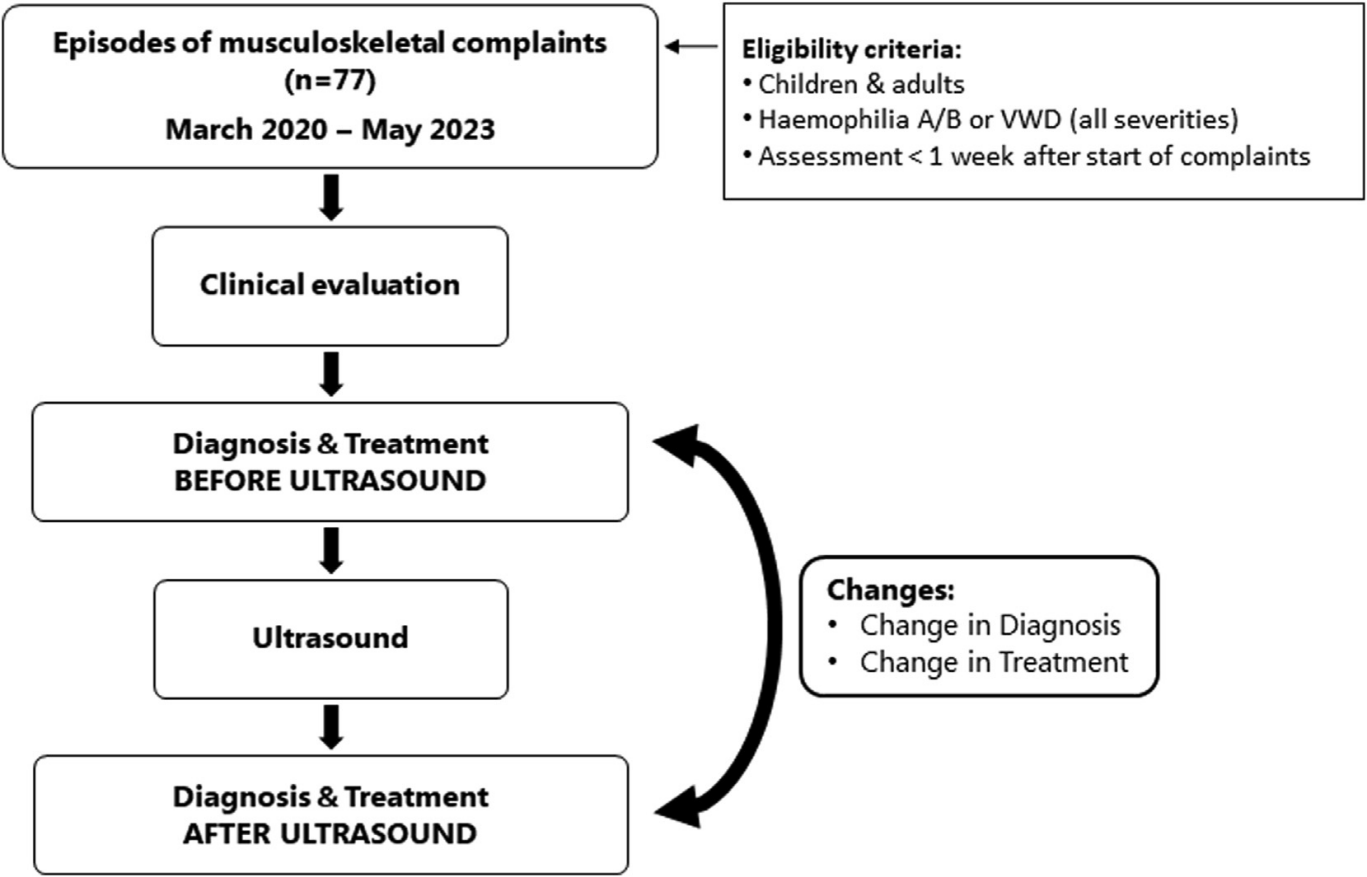
A flowchart summarizing the study design. VWD, von Willebrand disease.

The study design is summarized in Figure 1. This cross-sectional study included consecutive children and adults with hemophilia or VWD who attended our outpatient clinic with an acute musculoskeletal complaint between March 2020 and May 2023. Patients were included if they attended the outpatient clinic within 1 week of the onset of symptoms. Participants could contribute multiple episodes of musculoskeletal complaints. These could be complaints in different joints or muscles or complaints in the same joints or muscles when the previous complaints were considered fully recovered by the multidisciplinary team. An a priori sample size of at least 60 episodes was planned based on feasibility. No actual sample size calculation was performed.

For all episodes, participants first underwent clinical assessment. Based on the clinical assessment, an initial diagnosis and treatment plan were recorded on a case report form (CRF). Immediately afterwards, participants were assessed using point-of-care ultrasound. After the ultrasound, the final diagnosis and treatment plan were reported on the CRF. Clinical assessment and ultrasound assessment were both performed in the outpatient clinic during a single visit on the same day. Therefore, the ultrasound assessment did not significantly delay the start of treatment. Ethical approval for the study was waived by the Institutional Medical Ethical Review Board (20-089/C) as ultrasound and clinical assessment were part of daily clinical practice at our center. All patients gave informed consent for use of their data.

### 2.2 Clinical assessment

The clinical assessment consisted of history and physical examination and was performed by physicians and physiotherapists (M.A.T and J.B.) of our hemophilia treatment center (HTC). Established symptoms to differentiate between joint bleeds and arthropathy flare-ups [27] were collected: the cause of the musculoskeletal complaints (trauma/overexertion/unknown), pain (yes/no), pain localization (local/diffuse), type of pain (stabbing/pressing), pain at rest (yes/no), sleep disruption due to pain (yes/no), response of pain to treatment with factor replacement therapy (yes/no), pain with active range of motion (AROM; yes/no), and the course of the pain (constant/increasing/increasing with motion/start pain decreasing with motion). For lower extremity episodes, participants were additionally asked about pain on weight-bearing (yes/no).

The physical examination included assessment of swelling (yes/no), swelling localization (local/diffuse), discoloration (none/red/blue), warmth on palpation (yes/no), and AROM. AROM and swelling were assessed according to the Hemophilia Joint Health Score (HJHS) 2.1 [[28], [29], [30]]. For lower extremity episodes, participants’ gait (no weight-bearing/asymmetric gait/limited stability/no abnormalities) was also assessed. All the results of the clinical assessment were reported on the CRF.

### 2.3 Ultrasound assessment

Joint and/or muscle ultrasound was performed immediately after clinical assessment by 2 physiotherapists (M.A.T. and J.B.) in the outpatient clinic of our HTC. Both physiotherapists were trained and experienced in point-of-care musculoskeletal ultrasound in hemophilia care. Ultrasound assessments were performed using an Esaote MyLab 25 Gold ultrasound scanner with a 7.5- to 12-MHz linear transducer or a Mindray TE7 ultrasound scanner with a 6 to 14 MHz linear transducer. For joint episodes, effusion, synovial hypertrophy, and synovial hyperemia were assessed. For muscle episodes, fluid collection was assessed. Both joint effusion and muscle fluid collection were graded as 0 (absent/minimal), 1 (moderate), and 2 (large). Synovial hypertrophy in joints was graded as 0 (absent/minimal), 1 (mild/moderate), or 2 (severe) according to the Haemophilia Early Arthropathy Detection with Ultrasound (HEAD-US) protocol [31]. Synovial hyperemia in joints was assessed by power Doppler and graded as 0 (no signal), 1 (small spots), 2 (confluent vessels in <50% tissue of interest), or 3 (confluent vessels in ≥50% tissue of interest) according to the Joint Tissue Activity and Damage Examination ultrasound protocol [32]. All ultrasound findings were reported on the CRF.

### 2.4 Definitions of diagnosis and treatment

Before and after ultrasound assessment, the diagnosis and treatment were determined and reported on a CRF for all episodes. Diagnoses were categorized as one of the following options: intra-articular bleeds (joint bleeds), intramuscular bleeds (muscle bleeds), arthropathy, synovitis, distortion, tendinopathy/tendinitis, muscle tear/strain, or other diagnosis.

For the treatment plans, we recorded factor replacement therapy (dose and duration in days), prescription of anti-inflammatory medication (yes/no), mobilization advice (unload/limited loading/limit intensive loading/mobilization guided by pain/no mobilization restriction), follow-up (in days), and referral to a healthcare professional (primary care physiotherapist/orthopedic surgeon/both). Referral for additional imaging was also recorded.

### 2.5 Data collection and analysis

Age, gender (male/female), disease type and severity, use of prophylaxis, inhibitor status, bleeding episodes in the year prior to inclusion, and the HJHS closest to inclusion were extracted from the electronic patient records. For joint episodes in which recent joint-specific HJHS scores were not available, the osteochondral status of the joint was retrospectively estimated based on the ultrasound images or previous x-rays. Osteochondral status on ultrasound was scored according to the HEAD-US score [31] by 2 trained observers (F.H.P.v.L. and M.A.T.). X-rays were scored according to the Pettersson score [33] by a radiologist with >10 years of experience in imaging of hemophilic arthropathy (W.F.).

Patient, joint, and episode characteristics were reported as medians with IQRs for continuous variables and as frequencies with percentages for categorical variables.

Diagnosis and treatment before and after ultrasound were compared, and the proportion of changes with Clopper-Pearson “exact” 95% CIs was determined.

Changes in diagnoses before and after ultrasound were visualized in a Sankey diagram.

Additionally, the number needed to scan (NNS) for a change in diagnosis or treatment was calculated. The NNS describes how many patients need to be scanned by ultrasound in order to change the diagnosis or treatment for 1 patient [34]. The NNS was calculated as $NNS=\frac{1}{\text{absolute}\;\text{risk}\;\text{reduction}\;}$, where the absolute risk reduction was the proportion of changed diagnoses or treatment plans. Furthermore, the prevalence of misdiagnosis in “healthy joints” vs arthropathic joints (HJHS/HEAD-US cartilage and bone sum score/Pettersson score, >0) and children (aged <18 years) vs adults was compared using Fisher’s exact test. To examine the effect of patients contributing multiple episodes, a sensitivity analysis was performed that included only the first episode of each patient.

Finally, to explore the diagnostic value of clinical symptoms in identifying joint bleeding, the positive predictive value and negative predictive value (NPV) with CIs were calculated for clinical symptoms associated with joint bleeding. All analyses were performed in RStudio 2023.09.1+494 (Posit Software).

## 3 Results

### 3.1 Patients and episodes

Characteristics of the patients and acute musculoskeletal episodes are available in Table 1. We assessed 77 acute musculoskeletal episodes in 67 patients. Five patients were included twice with joint episodes in different joints. Two patients were included twice with 2 distinct joint episodes in the same joint. One patient was included 4 times with 2 muscle episodes in different muscles and 2 joint episodes in the right ankle.

**Table 1:** Patient and episode characteristics.

	Median or *n*	IQR or %
**Patients**	**(*****N*****= 67)**	
Age (y)	24	13-42
Children (<18 y)	27	40
Adults	40	60
Male	66	99
Disease		
Hemophilia A	57	85
Hemophilia B	6	9
von Willebrand disease	4	6
Hemophilia severity		
Severe	26	39
Moderate	12	18
Mild	25	37
Prophylactic treatment^a^	31	46
Age at the start of prophylaxis	3.6	1.7-11.6
Emicizumab	11	16
Positive inhibitor status	2	3
Bleeding episodes ≤1 y	1	1-3
**Episodes**	**(*****N*****= 77)**	
Extremity		
Upper	15	19
Lower	62	81
Location		
Joint	61	79
Muscle	11	14
Other	5	6
Cause		
Trauma	42	55
Overexertion	13	17
Unknown	22	29

Percentages might not add up to 100% due to rounding.

a Including 1 patient with 2 episodes who received on-demand treatment when included with the first episode and received prophylaxis when included with the second episode.

The study cohort included 34 episodes in children and 43 episodes in adults. The median age at the time of assessment was 24 years (IQR, 13-42 years). Most patients had hemophilia (94%). A total of 25 of 26 people with severe hemophilia, 3 of 12 people with moderate hemophilia, and 3 of 25 people with mild hemophilia received prophylactic treatment.

Approximately half of the musculoskeletal episodes (55%) were traumatic. Most episodes involved joints (79%), followed by muscles (14%). The majority of joint episodes (42/61, 69%) occurred in joints without signs of hemophilic arthropathy (HJHS, 0; HEAD-US cartilage and bone sum score, 0; Pettersson score, 0). In 40 of 61 joint episodes, a joint-specific HJHS score was available from the patient records, with a median of 1 year (IQR, 0-2 years) between the HJHS score and study inclusion. The median HJHS joint score for these joints was 0 (IQR, 0-1). Osteochondral status in 19 of the 21 remaining joints could be estimated retrospectively from ultrasound (*n* = 16) or previous x-rays (*n* = 3). The median sum of the HEAD-US cartilage and bone score was 0 (IQR, 0-1). X-rays showed 2 “healthy joints” (Pettersson score, 0) and 1 joint with hemophilic arthropathy (Pettersson score, 11). For only 2 joints, HJHS scores were not available and osteochondral status could not be estimated retrospectively.

### 3.2 Clinical impression

The clinical symptoms that patients presented with during the 77 acute musculoskeletal episodes are summarized in Supplementary Table S1. Pain (99%), painful AROM (90%), limited AROM (82%), and swelling (82%) were the most common symptoms. In addition, weight-bearing was painful in almost all lower-limb episodes (59/62, 95%). Based on the clinical symptoms only, joint bleeding was suspected in 37 episodes, muscle bleeding in 13 episodes, arthropathy in 4 episodes, synovitis in 1 episode, and other diagnoses in 22 episodes. A detailed list of suspected diagnoses before ultrasound based on the clinical impression is available in Table 2.

**Table 2:** Diagnoses before and after ultrasound assessment (*N* = 77).

Diagnosis	Before ultrasound *n* (%)	After ultrasound *n* (%)
Joint bleed	37 (48)	33 (43)
Muscle bleed	13 (17)	11 (14)
Arthropathy	4 (5)	2 (3)
Synovitis	1 (1)	1 (1)
Muscle, tendon, ligament injuries	16 (21)	20 (26)
Distortion	15 (19)	18 (23)
Tendinopathy/tendinitis	1 (1)	1 (1)
Muscle tear/strain	0 (0)	1 (1)
Other	6 (8)	10 (13)
Subcutaneous bleed	3 (4)	4 (5)
Trauma without bleeding	1 (1)	2 (3)
Overexertion without anatomical damage	1 (1)	2 (3)
Sinus tarsi syndrome	1 (1)	0 (0)
Thrombophlebitis	0 (0)	1 (1)
No diagnosis	0 (0)	1 (1)^a^

Percentages might not add up to 100% due to rounding.

a Unclear diagnosis based on ultrasound, either synovitis or arthropathy.

### 3.3 Ultrasound findings

Ultrasound findings are summarized in Supplementary Table S2. Joint effusion or muscle fluid collection was present on ultrasound in 44 of 77 episodes. Synovial hypertrophy was observed in 9 joints, and in 6 of them synovial hyperemia was visible with power Doppler. Ultrasound findings confirmed clinically suspected joint bleeding in 24 episodes. An additional 9 episodes were reclassified as joint bleed based on ultrasound findings. Ultrasound findings confirmed clinically suspected muscle bleeding in 9 episodes and diagnosed 2 additional muscle bleeds. Furthermore, after the ultrasound, 2 cases of arthropathy-related pain, 1 case of synovitis, 20 muscle/tendon/ligament injuries, and 10 other diagnoses were established. A detailed list of final diagnoses after ultrasound is available in Table 2.

### 3.4 Changes in diagnosis

**Figure 2:**
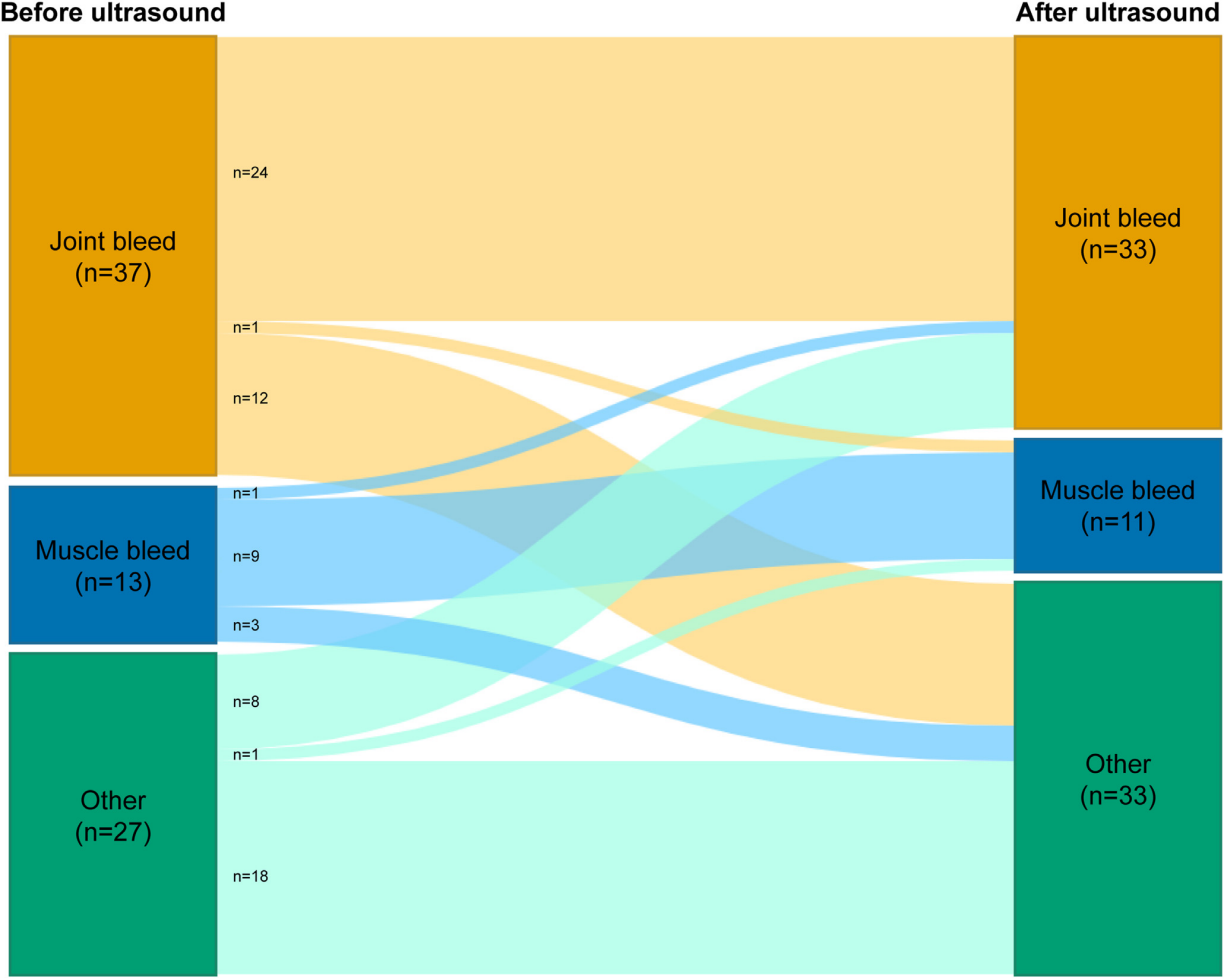
Sankey diagram visualizing the change in diagnoses before and after ultrasound assessment. Ultrasound findings changed the diagnosis in 28 of 77 episodes (36%; 95% CI, 26%-48%).

**Figure 3:**
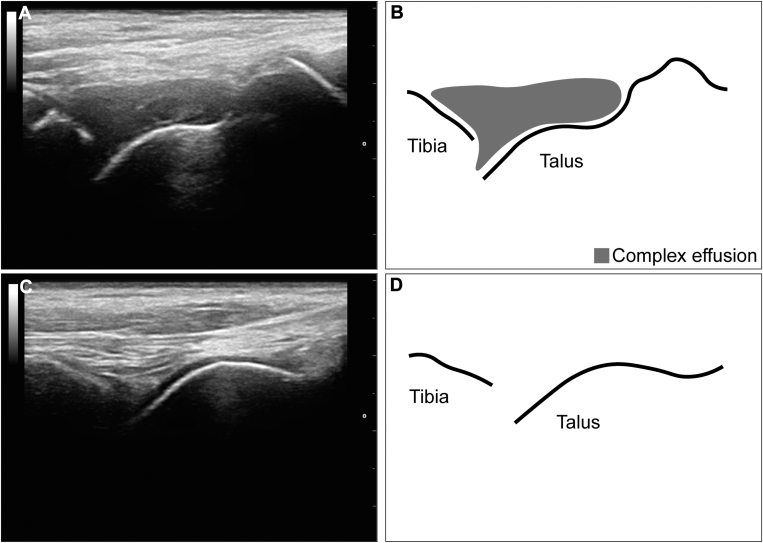
Midsagittal ultrasound images of the tibiotalar joint from 2 example cases illustrating discrepancies between clinical impression and ultrasound findings. Case 1: a traumatic, painful episode of the left ankle (Hemophilia Joint Health Score, 1) in a 10-year-old boy with severe hemophilia A on prophylaxis suspected of ligament injury based on clinical impression. After ultrasound, the final diagnosis was joint bleeding. The patient presented with constant diffuse pressing pain on weight-bearing and active range of motion (AROM). Despite the pain, AROM and gait were normal. Pain decreased after clotting factor concentrate. The patient had no pain at rest or during sleep. The ankle was not swollen and had a normal color and temperature. (A) Ultrasound showed complex joint effusion. (B) Graphic representation of the anatomical bony landmarks (distal tibia and talus) and the complex effusion on the ultrasound image in panel A. Case 2: a traumatic, painful episode of the left ankle (Hemophilia Joint Health Score, 0) in a 15-year-old boy with moderate hemophilia A treated on demand, suspected of joint bleeding based on clinical impression. After ultrasound, the final diagnosis was ligament injury. The patient presented with localized stabbing pain on weight-bearing and AROM. AROM was limited, and gait was asymmetric. Pain decreased with motion and after clotting factor concentrate. The patient had no pain at rest or during sleep. The ankle was diffusely swollen with normal color and temperature. (C) Ultrasound showed no joint effusion. (D) Graphic representation of the anatomical bony landmarks (distal tibia and talus) on the ultrasound image in panel C.

The diagnoses before and after ultrasound assessment are visualized in Figure 2 and summarized in Table 2. The ultrasound findings changed the diagnosis in 28 of 77 musculoskeletal episodes (36%; 95% CI, 26%-48%). Therefore, the NNS for a change in diagnosis was 3 musculoskeletal episodes. Based on the clinical impression alone, 9 musculoskeletal bleeds would have been missed, and in 2 episodes the type of bleeding would have been misclassified. In addition, 15 episodes would have been incorrectly treated as musculoskeletal bleeding. Misdiagnosis occurred with a similar prevalence in healthy joints (38%; 95% CI, 24%-54%) vs arthropathic joints (29%; 95% CI, 10%-56%; *P* = .76) and children (47%; 95% CI, 30%-65%) vs adults (28%; 95% CI, 15%-44%; *P* = .10). Patients contributing multiple episodes had little effect on the results, as the proportion of misdiagnoses was similar (34%; 95% CI, 23%-47%) when only the first episode of each patient was analyzed. Figure 3 shows ultrasound images from 2 example cases where ultrasound findings contributed to a change in diagnosis.

### 3.5 Change in treatment

**Figure 4:**
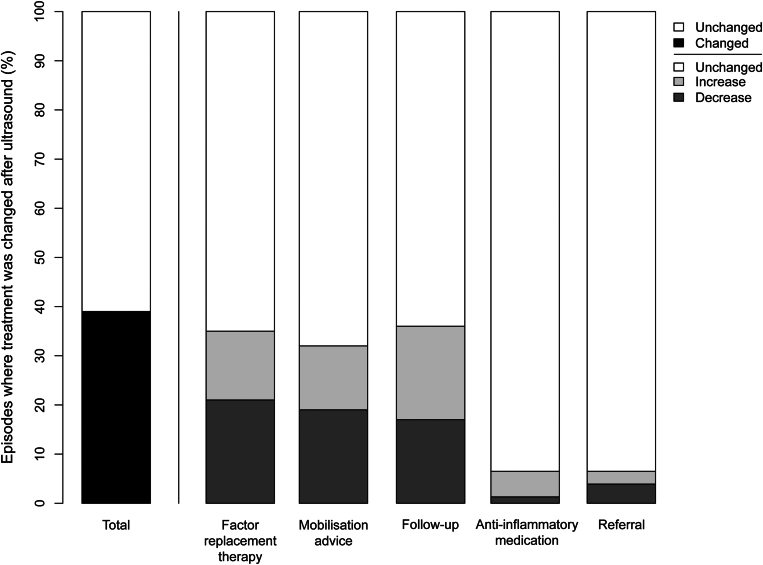
Stacked bar chart visualizing changes in treatment plans after ultrasound assessment.

Changes in treatment plans after ultrasound are visualized in Figure 4 and summarized in Supplementary Table S3. The ultrasound findings led to treatment alterations in 30 of 77 episodes (39%; 95% CI, 28%-51%). This corresponded to an NNS of 3 for any type of treatment alteration. The dose and/or duration of the factor replacement therapy was adjusted in 27 of 77 episodes (35%; 95% CI, 25%-47%). The total amount of international units of factor prescribed decreased in 16 episodes and increased in 11 episodes. The net effect was a reduction (total −96,879 IU) of the total international units of factor prescribed in the study population. The NNS for a change in dose and/or duration of factor replacement therapy was 3. Furthermore, changes in follow-up (28/77; 36%; 95% CI, 26%-48%) and mobilization advice (25/77; 32%; 95% CI, 22%-44%) were the most common. Ultrasound findings led to more restrictive mobilization advice in 15 episodes and more liberal mobilization advice in 10 episodes. Based on the ultrasound findings, prescription of anti-inflammatory medication changed in 5 episodes: 1 refrain from prescription and 4 extra prescriptions. Referral to a primary care physiotherapist was thought to be unnecessary in 3 episodes. On the contrary, additional referrals to a primary care physiotherapist were made in 2 episodes. In 8 episodes, patients were referred to the radiology department for an x-ray to rule out fracture (*n* = 3) or arthropathy (*n* = 1), an additional diagnostic ultrasound (*n* = 3), or both an additional diagnostic ultrasound and an x-ray to rule out fracture (*n* = 1). Changes in treatment plans occurred with a similar prevalence in healthy joints (38%; 95% CI, 24%-54%) vs arthropathic joints (41%; 95% CI, 18%-67%; *P* = 1) and children (47%; 95% CI, 30%-65%) vs adults (33%; 95% CI, 19%-49%; *P* = .24). Patients with multiple episodes had little effect on the results as the proportion of changes in treatment (37%; 95% CI, 26%-50%) and factor replacement therapy (33%; 95% CI, 22%-45%) after ultrasound was similar when only the first episode of each patient was analyzed.

### 3.6 Clinical symptoms for identifying joint bleeding

The prevalence of clinical symptoms in joint episodes (*n* = 61) according to the presence or absence of ultrasound-confirmed joint bleeding is summarized in Table 3. The sample size of our study did not allow detailed analyses of the predictive value of clinical symptoms for joint bleeding. However, preexisting hemophilic arthropathy seemed to be associated with joint bleeding. Besides preexisting hemophilic arthropathy, increasing pain intensity and diffuse pain seemed to have the highest positive predictive value for joint bleeding. The NPV was low for all 3 clinical signs. Traditional symptoms associated with joint bleeding, such as the presence of pain, limitation of AROM, and improvement of symptoms after factor concentrate treatment, were not associated with joint bleeding.

**Table 3:** Clinical symptoms in joint episodes with and without confirmed intra-articular bleeding.

Clinical symptom	Joint bleed (*n* = 33) *n* (%)	No joint bleed (*n* = 28) *n* (%)	PPV (95% CI)	NPV (95% CI)
Hemophilic arthropathy^a^	13 (39)	4 (14)	0.76 (0.56-97)	0.48 (0.33-0.63)
Cause				
Trauma	12 (36)	17 (61)		
Overexertion	8 (24)	3 (11)		
Unknown	13 (39)	8 (29)		
Pain	33 (100)	27 (96)		
Pain localization				
Local	13 (39)	21 (75)		
Diffuse	18 (55)	4 (14)	0.82 (0.66-0.98)	0.38 (0.22-0.55)
Not applicable	2 (6)	2 (7)		
Type of pain				
Stabbing	8 (24)	10 (36)		
Pressing	15 (46)	5 (18)		
Other	3 (9)	2 (7)		
Not applicable	7 (21)	10 (36)		
Pain in rest	17 (52)	10 (36)		
Sleep disrupted by pain^b^	12 (41)	3 (13)		
Painful weight-bearing^c^	23 (100)	24 (89)		
Painful AROM	30 (91)	23 (82)		
Pain decreased after FVIII treatment^d^	14 (61)	9 (53)		
Course of pain				
Constant	5 (15)	9 (32)		
Increasing	26 (79)	11 (39)	0.70 (0.56-0.85)	0.29 (0.11-0.47)
Increasing with motion	0 (0)	4 (14)		
Start pain (decreasing with motion)	2 (6)	4 (14)		
AROM limitation	27 (82)	20 (71)		
Warmth	22 (67)	12 (43)		
Swelling	31 (94)	19 (68)		
Local	10 (30)	11 (39)		
Diffuse	21 (68)	8 (42)		
Discoloration	4 (12)	4 (14)		
Red	0 (0)	2 (7)		
Blue	4 (12)	2 (7)		
Gait^c^				
No weight-bearing	6 (26)	5 (19)		
Asymmetric	12 (52)	15 (56)		
Limited stability	1 (4)	2 (7)		
No abnormalities	4 (17)	5 (19)		

Percentages might not add up to 100% due to rounding.

AROM, active range of motion; FVIII, factor VIII; NPV, negative predictive value; PPV, positive predictive value.

a Hemophilia Joint Health Score/Haemophilia Early Arthropathy Detection with Ultrasound cartilage and bone sum score/Pettersson score, >0.

b Applicable to 52 of 61 joint episodes as in 9 episodes, patients were assessed on the day of symptom onset.

c Only applicable to knee and ankle episodes (*n* = 50).

d Applicable to 40 of 61 joint episodes as patients received factor replacement therapy prior to assessment in only 40 of 61 episodes.

## 4 Discussion

In this prospective cross-sectional study, we evaluated the impact of ultrasound in addition to clinical assessment on the diagnosis and treatment of acute musculoskeletal episodes in children and adults with hemophilia and VWD, including joints with (29%) and without (67%) preexisting hemophilic arthropathy. Ultrasound findings impacted the diagnosis in 28 of 77 acute musculoskeletal episodes (36%; 95% CI, 26%-48%) and impacted treatment in 30 of 77 (39%; 95% CI, 28%-51%). This means that 3 patients need to be scanned by ultrasound in order to change either the diagnosis or the treatment of 1 patient (NNS, 3). Clinical symptoms evaluated in the current study were not able to identify joint bleeding.

### 4.1 Strengths and limitations

A strength of the current study is the relatively large heterogeneous study population. The mix of age, disease type and severity, and joint status provides a good representation of the daily variance in patients presenting with acute musculoskeletal complaints. Therefore, the results of our study provide a good impression of the impact of ultrasound in the daily practice of an HTC. Another strength of the study is the structured prospective data collection through the use of a CRF. This ensured data quality and reduced missing values. In addition, the structured collection of clinical symptoms on the CRF provided insight into the clinical decision-making process and ensured that clinicians made a well-grounded clinical diagnosis. Lastly, the ultrasound assessments were performed by 2 trained and experienced physiotherapists (M.A.T. and J.B.), which increased the accuracy of the ultrasound findings and reduced interobserver variability.

A limitation of the study is that only patients who visited the outpatient clinic with an acute musculoskeletal complaint were included. Therefore, patients without home treatment or home-treated patients who were unsure whether or not a bleeding event had occurred may be overrepresented in our study. The impact we observed is therefore representative of outpatient clinics in Western Europe, where self-infusion is common. Our results may not be fully generalizable to the home setting or to countries where self-infusion is not common. In addition, we did not collect data on the ethnicity of our patients. Therefore, we cannot comment on the influence of ethnicity on the generalizability of our results. However, we have no reason to believe that bleeding presents differently or that the bleeding frequency differs according to ethnicity. The study design has another limitation. The clinical assessment was always followed by an ultrasound, after which the final diagnosis and treatment were determined. This allowed the ultrasound assessment to act as a “safety net.” In cases of doubt based on clinical assessment, clinicians may have been more confident in their clinical diagnosis and treatment plan because they were aware that the final diagnosis was made and the treatment was planned after ultrasound. Our results may therefore underestimate the impact of ultrasound as clinicians, when unsure of their clinical diagnoses, are more likely to treat the episode as a bleed.

### 4.2 Comparison with previous literature

Three previous studies have investigated the value of ultrasound in diagnosis (and treatment) of acute painful musculoskeletal episodes in adults with hemophilia [14,24,25]. These reported changes in diagnosis after ultrasound in 16% (95% CI, 6%-32%) to 65% (95% CI, 48%-79%) of episodes, which is similar to the proportion of changed diagnoses in our study (36%; 95% CI, 26%-48%). Two of the previous studies reported changes in treatment after ultrasound in 16% (95% CI, 6%-32%) and 73% (95% CI, 56%-85%) [14,24]. The proportion of treatment changes in our study (39%; 95% CI, 28%-51%) was similar or lower.

Differences between our estimates and those of previous studies cannot be explained by the type of patients included. Previous studies investigated only musculoskeletal episodes in adults with hemophilia, including a large number of joints with preexisting hemophilic arthropathy. Our study included a large number of children (44% of episodes), and most joints were without hemophilic arthropathy. However, the prevalence of changes in diagnosis and treatment in the subgroup of adults and/or joints with hemophilia arthropathy was similar to our overall population.

Still, differences between our estimates and the previous results may be due to the smaller sample sizes of the previous studies (37-42 episodes) compared with our 77 episodes. Differences in setting (eg, HTC [14,25] vs home setting [24]) and differences in available resources (eg, prophylactic [24] vs on-demand treatment [25]) may have influenced the prevalence of musculoskeletal bleeding and thus the estimates. Furthermore, 2 studies included only patient-perceived hemarthroses [24,25], while our study and the study by Ceponis et al. [14] included all types of painful acute musculoskeletal episodes. Other differences were the time intervals between symptom onset and inclusion (previous studies, <72 hours; current study, <7 days) and the diagnostic outcomes used. The previous studies differentiated between bleeding and nonbleeding episodes. In our study, we divided the nonbleeding episodes into different diagnoses, which allowed for changes in diagnosis within the nonbleeding group. However, if we had only differentiated between bleeding and nonbleeding episodes, our results would not have been significantly different (diagnosis change in 24/77 episodes; 31%; 95% CI, 21%-43%).

### 4.3 Clinical relevance and future research

Our results showed that discrepancies between clinical diagnosis and ultrasound findings were common (36%; 95% CI, 26%-48%) in a heterogeneous group of patients presenting with acute musculoskeletal complaints. Based on clinical assessment alone, musculoskeletal bleeding would have been missed in 12% of episodes, 19% would have been incorrectly treated as musculoskeletal bleeding (overtreatment), and in 3%, the type of bleeding would have been misclassified (suboptimal treatment). These discrepancies demonstrate that ultrasound impacts diagnosis and treatment in patients with and without preexisting arthropathy. Ultrasound can improve the differentiation between joint bleeding and arthropathy-related complaints and between joint bleeding and nonhemophilia-related musculoskeletal complaints. Furthermore, the NPV of signs and symptoms derived from the literature for the detection of joint bleeding was low. This indicates that we are likely to miss bleeds based on clinical signs and symptoms alone.

In addition to the previously reported good accuracy compared with magnetic resonance imaging and reproducibility of ultrasound for musculoskeletal assessment in hemophilia [[18], [19], [20], [21]], we now show an impact of ultrasound on the management of acute musculoskeletal episodes. Ultrasound is noninvasive and relatively inexpensive and can be performed quickly by trained clinicians. We therefore encourage the use of ultrasound in addition to clinical assessment for the management of acute musculoskeletal complaints in people with bleeding disorders. Ultrasound may easily be incorporated into the standard diagnostic work-up for patients presenting to the outpatient clinic with an acute musculoskeletal complaint. In addition, patients could be encouraged to attend the clinic in case of a musculoskeletal complaint. Interestingly, there are several initiatives investigating the possibility of remote (artificial intelligence–assisted) ultrasound by patients at home or by nonhemophilia health professionals close to home [24,[35], [36], [37], [38], [39]]. In addition to increasing the accessibility of ultrasound, future research should focus on the (long-term) impact and cost-effectiveness of ultrasound-guided management of acute musculoskeletal complaints in people with bleeding disorders. To our knowledge, there is only limited research on the short-term effects of ultrasound-guided treatment [14,24]. Finally, the impact of ultrasound in addition to clinical assessment may be influenced by the prevalence of musculoskeletal bleeding in the patient population. Consequently, in the home setting or in HTCs in countries with different resources, the NNS may be different. Given that the results of the Uruguayan study [25], in which all patients were treated on demand, and the Spanish home-delivered ultrasound study [24] are similar to ours, we expect ultrasound to add value in such other settings as well. However, this is a topic for further research.

## 5 Conclusion

Frequent discrepancies between clinical diagnosis and ultrasound findings were observed in acute musculoskeletal episodes in a heterogeneous cohort of children and adults with hemophilia and VWD with and without hemophilic arthropathy. Ultrasound findings in addition to clinical assessment impacted diagnosis in 36% and treatment plans in 39% of episodes, which corresponds to an NNS of 3 for a change in diagnosis and/or treatment. Our results show that it is difficult to correctly diagnose an acute musculoskeletal episode based on clinical assessment in both joints with and without hemophilic arthropathy. We therefore encourage implementation of ultrasound in the management of acute musculoskeletal complaints in people with hemophilia and VWD.
