# Supplementary material for: Ultrasound in addition to clinical assessment of acute musculoskeletal complaints in bleeding disorders: impact on patient management
Source: Res Pract Thromb Haemost. 2024 Mar 15;8(2):102372. doi: 10.1016/j.rpth.2024.102372 (PMC10999475; doi:10.1016/j.rpth.2024.102372)
Supplement: Supplementary Material [file mmc1.docx]

**Supplementary Material**

| **Supplementary Table 1**. Clinical symptoms (n=77) | | |
| --- | --- | --- |
|  | n | % |
| **Pain** | 76 | 99% |
| **Pain localisation** |  |  |
| *Local* | *45* | *58%* |
| *Diffuse* | *27* | *35%* |
| *n.a.* | *4* | *5%* |
| **Type of pain** |  |  |
| *Stabbing* | *23* | *30%* |
| *Pressing* | *29* | *38%* |
| *Other* | *5* | *6%* |
| *n.a.* | *19* | *25%* |
| **Pain at rest** | 38 | 49% |
| **Sleep disrupted by pain^§^** | 21 | 27% |
| **Painful weightbearing^¶^** | 59 | 95% |
| **Painful AROM** | 69 | 90% |
| **Pain decreased after factor replacement therapy†** | 25 | 32% |
| **Course of pain** |  |  |
| *Constant* | *16* | *21%* |
| *Increasing* | *51* | *66%* |
| *Increasing with motion* | *4* | *5%* |
| *Start pain (decreasing with motion)* | *6* | *8%* |
| **AROM limitation** | 63 | 82% |
| **Warmth** | 44 | 57% |
| **Swelling** | 63 | 82% |
| *Local* | *29* | *38%* |
| *Diffuse* | *34* | *44%* |
| **Discoloration** | 15 | 19% |
| *Red* | *2* | *3%* |
| *Blue* | *13* | *17%* |
| **Gait**^¶^ |  |  |
| *No weightbearing* | *13* | *21%* |
| *Asymmetric* | *37* | *60%* |
| *Limited stability* | *3* | *5%* |
| *No abnormalities* | *9* | *15%* |
| % might not add up to 100% due to rounding, § Applicable to 68/77 episodes as in 9 episodes patients were assessed on the day of symptom onset, ¶ Only applicable to lower extremity episodes (n=62), †Applicable to 49/77 episodes as patients received factor replacement therapy prior to assessment in only 49/77 episodes, AROM: active range of motion | | |

| **Supplementary Table 2**. Ultrasound findings (n=77) | | |
| --- | --- | --- |
|  | n | % |
| **Effusion/fluid collection (n=77)** | 44 | 57% |
| *Minimal/moderate* | *10* | *13%* |
| *Large* | *34* | *44%* |
| **Synovial hypertrophy (n=70)^¶^** | 9 | 13% |
| *Mild/moderate* | *8* | *11%* |
| *Severe* | *1* | *1%* |
| **Synovial hyperaemia (n=68)^¶†^** | 6 | 9% |
| *Small spots* | *5* | *7%* |
| *Confluent vessel in <50% tissue of interest* | *1* | *1%* |
| % might not add up to 100% due to rounding; **¶ A**vailable for joint ultrasound only (n=70/77); †missing for 2 joints. | | |

| **Supplementary Table 3**. Treatment before and after ultrasound assessment (n=77) | | |  |
| --- | --- | --- | --- |
|  | Before ultrasound  Median/n (IQR/%) | After ultrasound  Median/n (IQR/%) | Change  n (%) |
| **Factor replacement therapy** | 58 (75%) | 50 (65%) | 27 (35%) |
| *Total dose (IU)* | 4000 (500-10000) | 3000 (0-8000) |  |
| *Duration (days)* | 3 (1-4) | 2 (0-4) |  |
| **Anti-inflammatory treatment** | 6 (8%) | 8 (10%) | 5 (6%) |
| **Mobilisation** |  |  | 25 (32%) |
| *Unload* | *33 (43%)* | *26 (34%)* |  |
| *Limited loading* | *18 (23%)* | *19 (25%)* |  |
| *limit intensive loading* | *5 (6%)* | *10 (13%)* |  |
| *Mobilisation guided by pain* | *19 (25%)* | *17 (22%)* |  |
| *No mobilisation restriction* | *2 (3%)* | *5 (6%)* |  |
| **Referral** |  |  | 5 (6%) |
| *Physiotherapist p.c.* | *12 (16%)* | *10 (13%)* |  |
| *Physiotherapist p.c. +*  *orthopaedic surgeon* | *0 (0%)* | *1 (1%)* |  |
| **Follow-up** | 49 (64%) | 46 (60%) | 28 (36%) |
| *Follow-up interval (days)* | 7 (7-7) | *7 (4-10)* |  |
| % might not add up to 100% due to rounding; Physiotherapist p.c.: referral to a primary care physiotherapist | | | |
